# Resisting epistemic loss in AI image generation

**DOI:** 10.1016/j.patter.2026.101568

**Published:** 2026-06-12

**Authors:** Abdullah Hasan Safir, Alan F. Blackwell, Ramit Debnath

**Affiliations:** 1 Collective Intelligence and Design Group, University of Cambridge, Cambridge, UK; 2 Department of Computer Science and Technology, University of Cambridge, Cambridge, UK; 3 Centre for Human-Inspired AI (CHIA), University of Cambridge, Cambridge, UK

## Abstract

Hintze et al.’s recent study highlights the tendency of current-generation vision-language models to converge on overly generic outputs. We argue that considering AI imageries as epistemic artefact and AI-driven artistic practices as socio-cultural processes can provide better understandings around the implications of such conditions beyond merely technical aspects of image generation. Drawing from our ongoing research, we highlight the necessity of bringing the epistemic vulnerability of marginalized artists and users and their hierarchical relations with these tools into sociotechnical design conversations, and by doing so, to explore the possibility for pluriversal and just AI futures, particularly in the Global South.

## Main text

In a recent paper, Hintze et al.^1^ show that automated AI image generator systems, specifically in prompt → image → prompt loops, keep remixing only a dozen repetitive, cliché motifs from stock photos on the internet. We find this study important and applaud its contributions, both in terms of its innovative method and critical implications of its findings.

For image generation, the researchers used Stable Diffusion XL, and for image description, they used Large Language and Vision Assistant. They built iterative feedback loops between the two and ran over 100 iterations with different initial prompts. The experiment resulted in generating bland, almost identical visuals—they compare it with boring “elevator music”—across 700 undertaken trajectories in 7 temperature settings. Such biases and redundancies may cause diversity collapse in the machine-generated creative landscape by reinforcing aesthetic and cultural uniformity.

Recent research by us and others from a socio-cultural perspective has identified similar patterns in AI-driven artistic production.^2^^,^^3^^,^^4^ These in-depth qualitative analyses suggest that AI-assisted image generation can unsettle human imagination and may ultimately erode something profound in the process: the rich, plural bodies of knowledge that have long sustained creative practices. In essence, this extends Hintze et al.'s highlighted harms in cultural and aesthetic domains toward epistemic realms, indicating reshaping, if not suppressing, the diversity of human knowledge systems by data-driven generative systems. As a result, proposed solution-centric approaches of human-AI collaboration in the paper may appear weaker, or even ineffective when we consider marginalized artists’ epistemic vulnerability and hierarchical relations with these AI tools, particularly of those who reside and practice in the Global South.

### AI imageries as epistemic artifact

**Figure 1:**
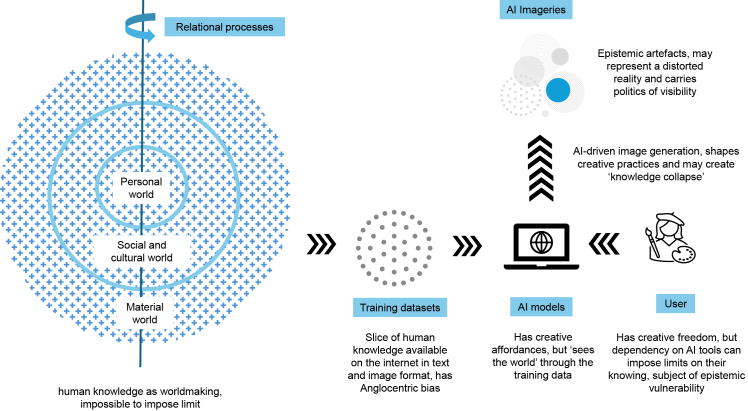
AI and the order of knowledge in image generation Source: authors.

Current text-to-image generation methods rely on models trained on massive image datasets to learn visual patterns, then use a diffusion process that begins with random noise and iteratively removes it during inference on a user’s prompt to produce a coherent image. By going beyond such technical understanding, we situate AI-generated imageries culturally to better understand their impacts and understand AI-driven artistic production from communities’ standpoints. When artists interact with generative AI tools for their creative practices, this does not create a stable order like the applications of previous non-automated creative digital tools where the control mostly remained in artists’ hands. Instead, generative AI tools function as creative “mediators,” transforming artists’ creative ideas into the digital form in partially autonomous way, and at the same time, they actively produce, circulate, stabilizes, and contest artists’ knowledge of their surrounding personal, social, and material world during artistic production (see Figure 1).

In their culture-centric study of text-to-image (T2I) models, Qadri et al.^2^ highlighted how their participants from India, Pakistan, and Bangladesh articulated the failure of these tools to generate cultural subjects in South Asian contexts and how they can amplify hegemonic cultural defaults and perpetuate cultural tropes. We read these findings as an indication of the models’ role as representational media. As the authors also highlight, while mirroring a distorted reality, AI tools can materially constrain users’ epistemic roles of inference, exploration of possibilities, and intervention through art.

Later, another group of human-computer interaction (HCI) researchers documented the challenges of low-literate, marginalized artists from Bangladesh to translate their creative ideas, linguistic metaphors, local art and architectural styles, context-representing visual dialects, and the notions of positive social transformation through their artworks while using generative AI tools.^3^ We understand that making art with AI functions as an “epistemic action”^5^—a situated, interactive process where tools can reorganize information, attention, senses, and eventually what we call “knowledge.”

Therefore, it is of immense value to understand AI images as “epistemic artifacts.” They embody and externalize representations, structure, constrain creative practices, and enable or destabilize epistemic work, such as sensemaking, and above all carry politics—altering who can know what, with what authority, and at what cost.

We use this analytical core in our ongoing works exploring social, cultural, and ethical implications of AI image generation, particularly in Global South contexts. In a recent project,^4^ we designed an exploratory human-AI interaction experiment with DALL-E.3. We systematically developed textual prompts drawing from both art history and prompt engineering domains and attempted to reproduce select artworks by a prominent decolonial Bangladeshi artist SM Sultan. Sultan’s art represents imaginations of the lower social class people and their everyday experiences, who are often marginalized in postcolonial societies like Bangladesh. In our study, we observed (1) severe misrepresentation of everyday cultural elements and objects of rural Bangladeshi populations groups in the resulting AI images, such as “boti,” “sari,” “lungi,” or “gamcha”; (2) an overshadowing of local aesthetic motifs, symbols, and styles; and (3) distortion of overall visual narratives that the original artworks wanted to communicate. Our experimental approach imitates marginalized artists’ efforts to solve these issues by providing long, detailed, or accurate prompts, using native languages or taking brainstorming help from ChatGPT (we even applied original image → prompt → AI image loop) but shows that in every case they struggle to obtain their desired accurate results.

We developed a carefully curated digital archive (see Safir et al.^4^) with the AI-generated artworks from the project and later, arranged a participatory engagement session with some Bangladeshi formally trained artists with this archive. During the focus group discussion, the artists told us that for many of them, art is not just a form of expression but a critical tool for voicing social issues, reclaiming histories, and sharing fresh perspectives. They shared personal accounts around how AI tools limit their creative imaginations in everyday uses, although they were not sure why and how—the archive exemplified what erasure of worldviews and knowledge meant to them. Later, we showcased the archive in an exhibition in Cardiff, Wales at BritCHI, the largest gathering of British HCI researchers. Scholars and practitioners were candid; they said that they rarely approached generative AI’s implications from such a marginal point of view, and they could sense how AI’s new worldmaking could unfold, where certain population groups can remain as “other,” with many knowledge systems around our current material world sidelined. Currently, in a working paper, we are synthesizing these insights to inform sociotechnical design conversations in and around generative AI.

To understand AI art as epistemic artifacts thus helps us imagine how AI can potentially transform knowledge practices of the artists, and at the same time, of aesthetes and general audience by structuring what can be noticed, inferred, and justified. Such framing helps situate AI-driven artistic practices as deeply embedded in social and cultural systems beyond their impact on individual users. AI art as epistemic artifacts also involve trade-offs; they can potentially compress reality in ways that reshape creative practices, while also introducing blind spots, path dependencies, and politics of visibility.

### Epistemic loss and Global South AI futures

Current AI models are trained with a tiny portion of humanity’s knowledge. In a project named “knowing machines,”^6^ researchers visually portray the geopolitical asymmetries in terms of demographic or cultural distribution in big image datasets (such as LAION-5B) reportedly used for training foundational models like Midjourney and Stable Diffusion. These datasets are built on either non-profit web infrastructures like Common Crawl or commercial platforms like Pinterest, Shopify, and stock presentation sites. Far from representing “ground truth,” such imageries are either influenced by concentration of internet infrastructure, content production, and hosting capacity or search engine optimization, consumer markets, design aesthetics, and retail cultures, both disproportionately privileging Global North and WEIRD (Western, educated, industrialized, rich, and democratic) contexts. Machine-readable alternative text and captions are necessary for image-text pairing, which favors English and other dominant languages. Images in underrepresented languages or with non-standardized metadata typically have poor indexing. Although they are scalable, automated filtering and similarity thresholds used in datasets such as LAION-5B also reinforce platform biases and unequal representation.

Hintze et al.^1^ also explained the convergence toward specific attractors in their study, for example, stormy lighthouses, gothic cathedrals, and palatial interiors, attributing this to the over-representation of Western-centric cultural and contextual biases in stock photos, tourist imagery, and commercially viable visual content found in internet-scale training corpora. However, they also draw parallels with human cultural transmission with such algorithmic drift, but when it literally stands upon uneven production and distribution of knowledge representations in training data, such a conclusion seems myopic. Andrew Peterson,^7^ however, provides a more convincing explanation by providing evidence of how AI models naturally produce output that is closer to the center of the distribution, even if they are trained on enormous volumes of heterogeneous inputs. Humans may deliberately seek out various types of information if they believe them to be valuable, while such models are unable to freely select from the data they are trained on. Peterson argues that a widespread dependence on recursive AI systems could result in “knowledge collapse,” a process that could be detrimental to innovation and the depth of human knowledge and culture.

Attention to Global South contexts shows that marginalized groups can be disproportionately affected by these dynamics. Here, the risks include not only economic or technical exclusion, but also epistemic losses: the erosion of worldviews, ontologies, and ways of knowing under the pressure of AI’s largely Western-centric assumptions. This perspective highlights the complexity of human-AI collaboration in creative work, given a long history in which Western art was treated as the standard or norm and Eastern art was portrayed as exotic, backward, or inferior, valued only when it conformed to or influenced Western aesthetics (see Safir et al.^4^). Contemporary AI art-making practices risk reproducing similar patterns of dominance, creating epistemic vulnerability for Global South artists by constraining their artistic imaginations.

### Call to action for epistemic justice

Here, we call scholars and practitioners to consider the Global South not only as a site where epistemic injustices in AI are reproduced, but also as a site of possibility for pluriversal and just AI futures. A recent work (led by the first author) with diverse artist communities in Bangladesh, including rickshaw painters, street artists, and henna designers,^8^ demonstrates how practices of cultural resonance, co-creation, and mutual recognition can sustain collective agency. These practices shape relationships with local people, materials, and artistic traditions in ways that exceed the narrow framings of AI-as-tool or artist-as-individual creator. Building on such insights, future AI research, design, and policy should actively incorporate artists’ solidarities, community-centric data stewardship, and collective bargaining mechanisms into the ethical development of AI tools so that artists can reclaim control over their creative practices and contest how AI technologies abstract, disembed, or commodify art-making and challenge individual-centric ethical standards that dominate Western discourses.

We therefore urge AI researchers, developers, and institutions to move beyond solutionist prescriptions, such as larger datasets or more powerful models, as the default response to epistemic asymmetries in AI image generation. In a hyper-unequal world where data infrastructures are largely owned and governed by Northern corporations and institutions, communities in the Global South are repeatedly positioned as data subjects without meaningful say over how their images are collected, labeled, circulated, or monetized. Instead of designing governance mechanisms from the top down, detached from situated knowledge practices, AI scholarship and industry practice should prioritize processes that are built with communities from the ground up. This entails reorienting research agendas toward what these communities know, how they know it, and how AI tools might support rather than overwrite or instrumentalize their ways of knowing through artistic practices.

## Declaration of interests

The authors declare no competing interests.

## Declaration of generative AI and AI-assisted technologies in the manuscript preparation process

During the preparation of this work the authors used Microsoft Co-pilot in order to lightly edit some texts. After using this tool, the authors reviewed and edited the content as needed and take full responsibility for the content of the published article.
